# Does Duty Cycle Modification Affect Temperature During Thulium Fiber Laser Lithotripsy?

**DOI:** 10.1002/lsm.70067

**Published:** 2025-10-26

**Authors:** Kallan Richards, Katya Hanessian, Ali Albaghli, Gabriel Martin, Daniel Jhang, Adel Battikha, Joshua Ghoulian, Zham Okhunov, D. Duane Baldwin

**Affiliations:** ^1^ Department of Urology Loma Linda University Health Loma Linda California USA

**Keywords:** lithotripsy, Laser, nephrolithiasis, temperature, urinary Calculi, urolithiasis

## Abstract

**Objectives:**

Although the thulium fiber laser (TFL, 1940 nm) efficiently fragments stones, concerns have been raised regarding heat production. Recently, a TFL with a modified duty cycle (MDC) was designed to reduce heat generation. The purpose of this study was to compare heat generation between the MDC and conventional TFL.

**Methods:**

Ten millimeter BegoStone phantoms were placed in the proximal ureter of a kidney and ureter model. The model was submerged in a 35°C saline bath, with continuous saline irrigation (22°C) maintained at 15 mL/min through a ureteroscope. Temperature was measured using a needle thermocouple. Five trials of 60 s continuous laser activation were performed for each power setting: 3 W (0.3 J/10 Hz), 10 W (1 J/10 Hz), 20 W (1 J/20 Hz) and 30 W (0.6 J/50 Hz). Thermal dose was calculated as cumulative equivalent minutes at 43°C (CEM₄₃; injury threshold> 20). Kruskal‐Wallis and Mann‐Whitney U tests were used for statistical analysis with *p* < 0.05 being significant.

**Results:**

Both lasers produced maximum temperatures (*T*_max_) of 27°C at 3 W. The MDC TFL generated a significantly lower Tmax than the conventional TFL at 10 W (36.9°C vs 42.1°C, *p* < 0.001), 20 W (38.1°C vs 44.6°C, *p* < 0.001), and 30 W (52.5°C vs 63.6°C, *p* < 0.05). CEM₄₃ for the MDC and conventional TFL at 20 W were (0.00 vs 0.90, respectively), and at 30 W (425 vs 275,919, respectively).

**Conclusions:**

The MDC TFL generated lower temperatures at 10, 20, and 30 W. Future studies are necessary to evaluate stone fragmentation efficiency of the MDC TFL.

**Abbreviations**
CT: computed tomography
HL: holmium laser
IDC: intrinsic uuty cycle
MDC: modified duty cycle
ODC: operative duty cycle
PDC: procedural duty cycle
TFL: thulium fiber laser
Tmax: maximum temperature
URS: ureteroscopy

## 1 Introduction

Urolithiasis remains a significant health concern with rising incidence, impacting 1 in 11 individuals in the United States, with at least 50% recurrence within 10 years [1]. Retrograde intrarenal surgery (RIRS) with laser lithotripsy is the preferred surgical modality for management of kidney and ureteral stones smaller than 1.5 cm [2]. Over the past decade, numerous innovations in RIRS have made it one of the fastest‐growing fields in urology. Advancements in laser technology have revolutionized the treatment of urolithiasis. Currently, two major laser platforms are available in the market: the holmium laser (HL) and the thulium fiber laser (TFL). Numerous studies have compared these modalities, highlighting the advantages and limitations of each.

Since it operates at 1940 nm, the TFL has a higher water absorption coefficient than the HL, increasing its efficiency in fragmenting stones [3]. However, despite its ability to effectively break stones, the Soltive TFL generates more heat, potentially leading to increased risk of irreversible cell damage, protein denaturation, and tissue necrosis [4, 5]. Several factors have been explored to mitigate the heat effects of the TFL, including various laser settings, irrigation rates, and irrigation temperatures [6, 7]. Recently, modifying the laser duty cycle (MDC) has been proposed as a potential solution to reduce heat generation during the TFL lithotripsy. The purpose of this study was to evaluate the temperature profile of the TFL with an MDC and compare it to the conventional TFL platform during laser lithotripsy.

## 2 Methods

**Figure 1:**
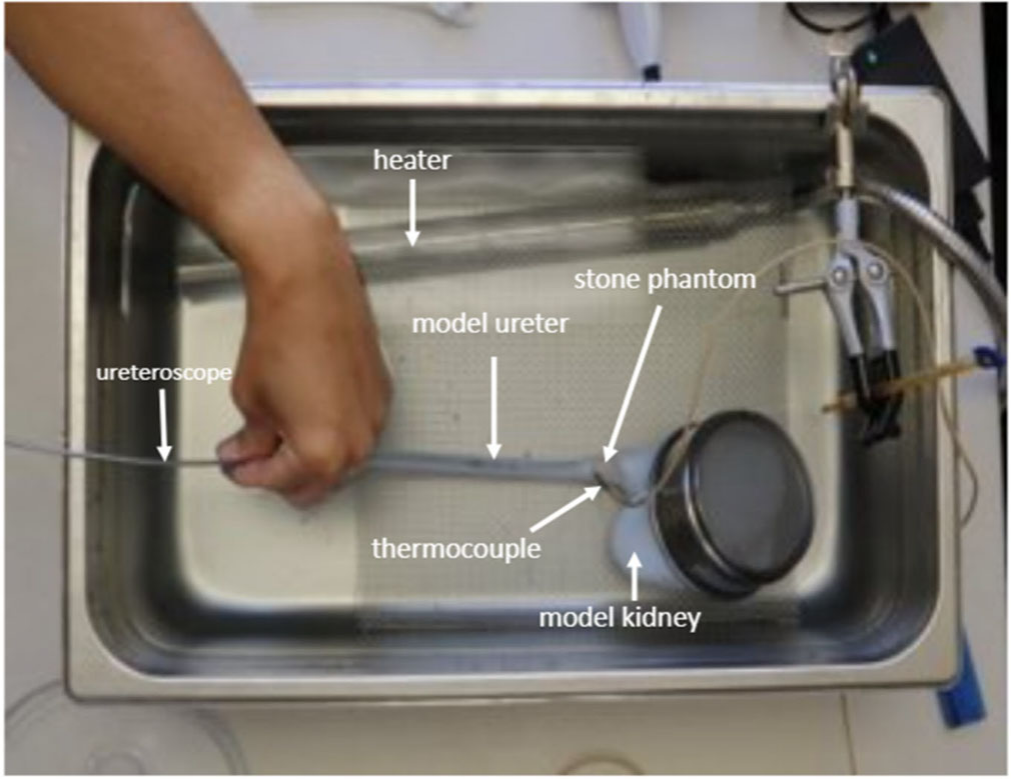
Experimental setup.

In this study, a bench top model was created to compare the temperature profiles generated by the conventional Soltive SuperPulsed laser (Olympus, Center Valley, PA) to the MDC OPTICA XT laser (Convergent, Oakland, CA). A kidney model was constructed based on a patient's computed tomography (CT) imaging using 3D Slicer 5.2.2 (Kitware, NY), while the ureter was designed with Autodesk Fusion 360 (Autodesk, San Francisco, CA). The models were created using molds made of polylactic acid (3D Universe, Algonquin, IL) with an Ultimaker 3 Extended (Ultimaker, Geldermalsen, Netherlands) 3D printer and coated with liquid Dragon Skin 20 silicone (Smooth‐On Inc., Macungie, PA) to simulate tissue properties. Identical 10 mm spherical BegoStones (Bego GmbH, Bremen, Germany) with a calcium oxalate monohydrate composition were created to replicate ureteral stones. These stones were submerged in a saline bath for 24 h before each trial and positioned at a 2 cm distal to the ureteropelvic junction to simulate an impacted proximal ureteral stone (Figure 1).

The kidney model was submerged in a 35°C saline bath using an electric immersion heater (Pong‐dang, Gyeonggi‐do, South Korea) to maintain core temperature during endoscopic surgery. To monitor ureteral fluid temperatures, a type T needle thermocouple (ThermoWorks, American Fork, UT) was placed in the ureteral wall 2 mm lateral to the 200 µm core laser fiber tip, within the region of laser irradiation. A digital data acquisition system, including a MAX31855 T‐Type Thermocouple Sensor breakout board (ThermoWorks, American Fork, UT) recorded temperature data with readings every 6.0 s. The Thermedx FluidSmart System (Thermedx LLC, Solon, OH) was used to maintain continuous saline irrigation at 15 mL/minute at room temperature (22°C). The 200 µm core laser fiber was advanced through a 7.5 Fr single‐use digital flexible ureteroscope (Pusen Medical Technology, Zhuhai, China) that was inserted into the model ureter until the laser fiber contacted the stone. All trials were conducted with lasers operating in long pulse mode (0.5–10 ms).

Intraluminal temperatures were recorded during 60 s of continuous laser activation at 1940 nm for each specified power setting: 3 W (0.3 J/10 Hz), 10 W (1 J/10 Hz), 20 W (1 J/20 Hz) and 30 W (0.6 J/50 Hz). The order of the lasers and power settings was randomized, with five trials performed for each power setting. The time to reach the maximum temperature (Tmax) was recorded. Cumulative thermal dose was calculated as cumulative equivalent minutes at 43°C (CEM₄₃), using the equations established by Sapareto and Dewey [4]. The threshold for thermal injury was defined as CEM₄₃ greater than 20, while instantaneous tissue damage occurs at 60°C [5].

The analysis included a comparison of average and maximum temperatures of ureteral fluid between the two laser systems at each power output. Statistical analyses were performed using SPSS Statistics 27 (IBM, Chicago, IL), with a significance threshold set at *p* < 0.05. Kruskal‐Wallis and Mann–Whitney U tests were performed to compare temperature and CEM₄₃ values at each setting.

## 3 Results

**Figure 2:**
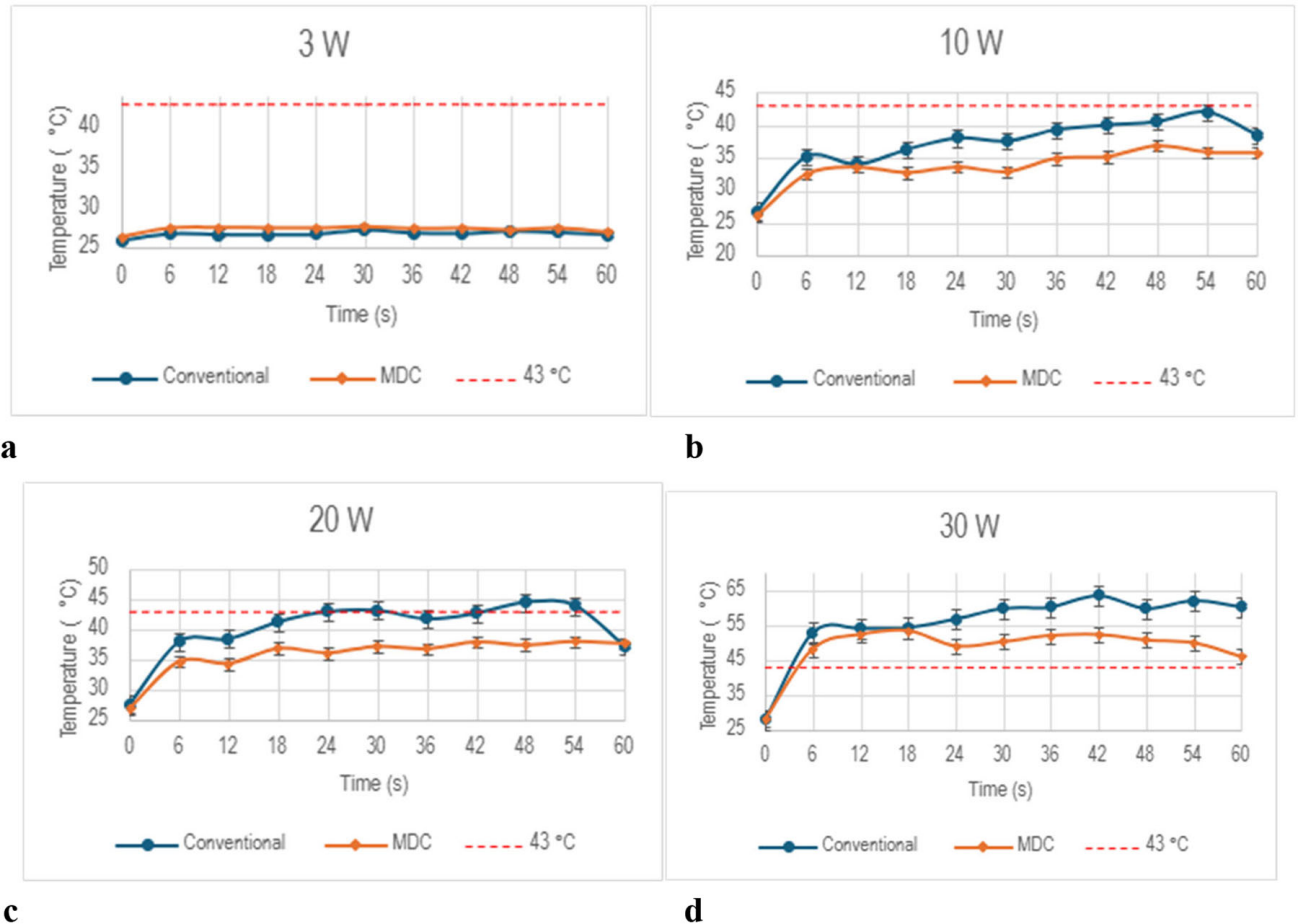
Temperature curves for the conventional TFL and MDC TFL at 3 W (a), 10 W (b), 20 W (c), 30 W (d).

**Figure 3:**
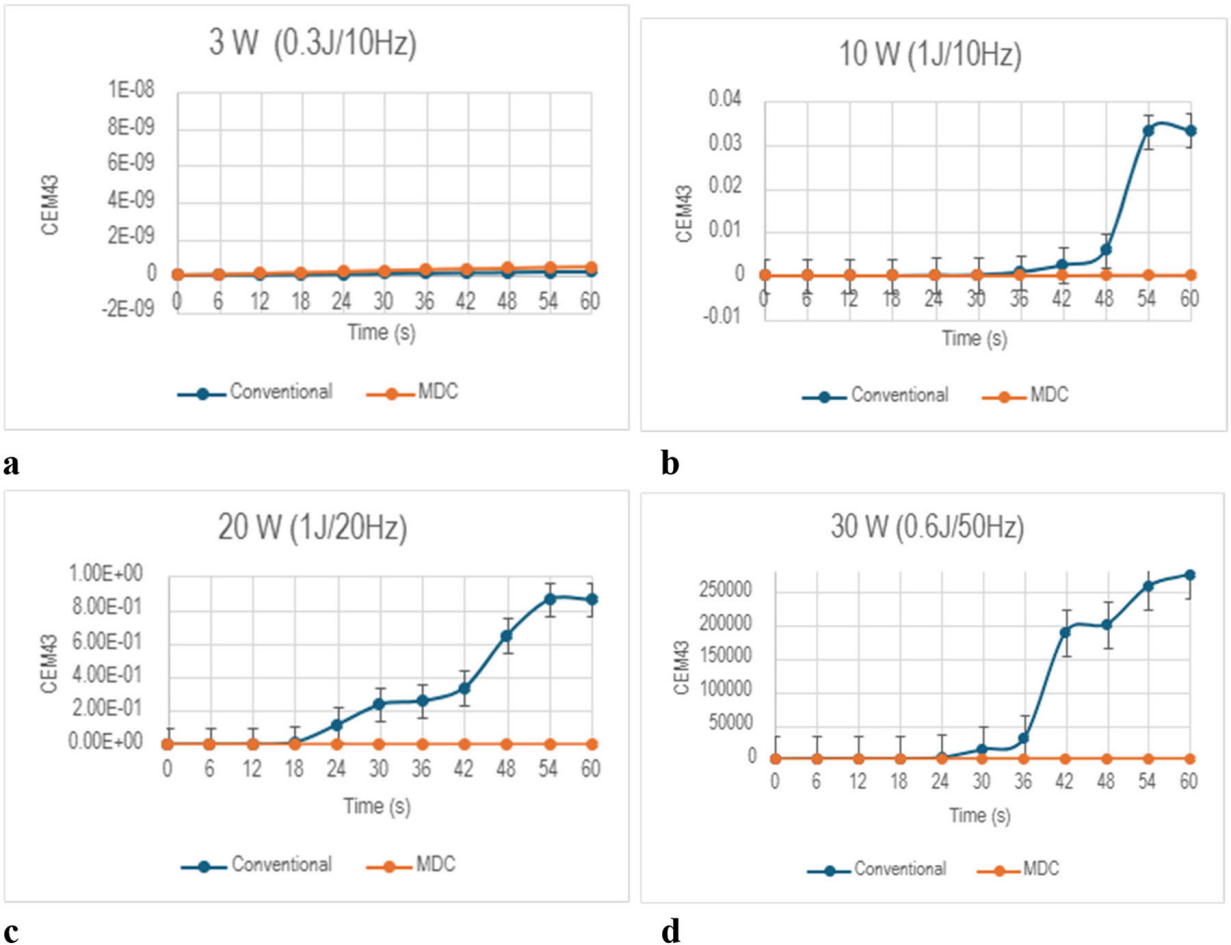
CEM₄₃ curves for the standard TFL and MDC TFL at 3 W (a), 10 W (b), 20 W (c), 30 W (d).

**Figure 4:**
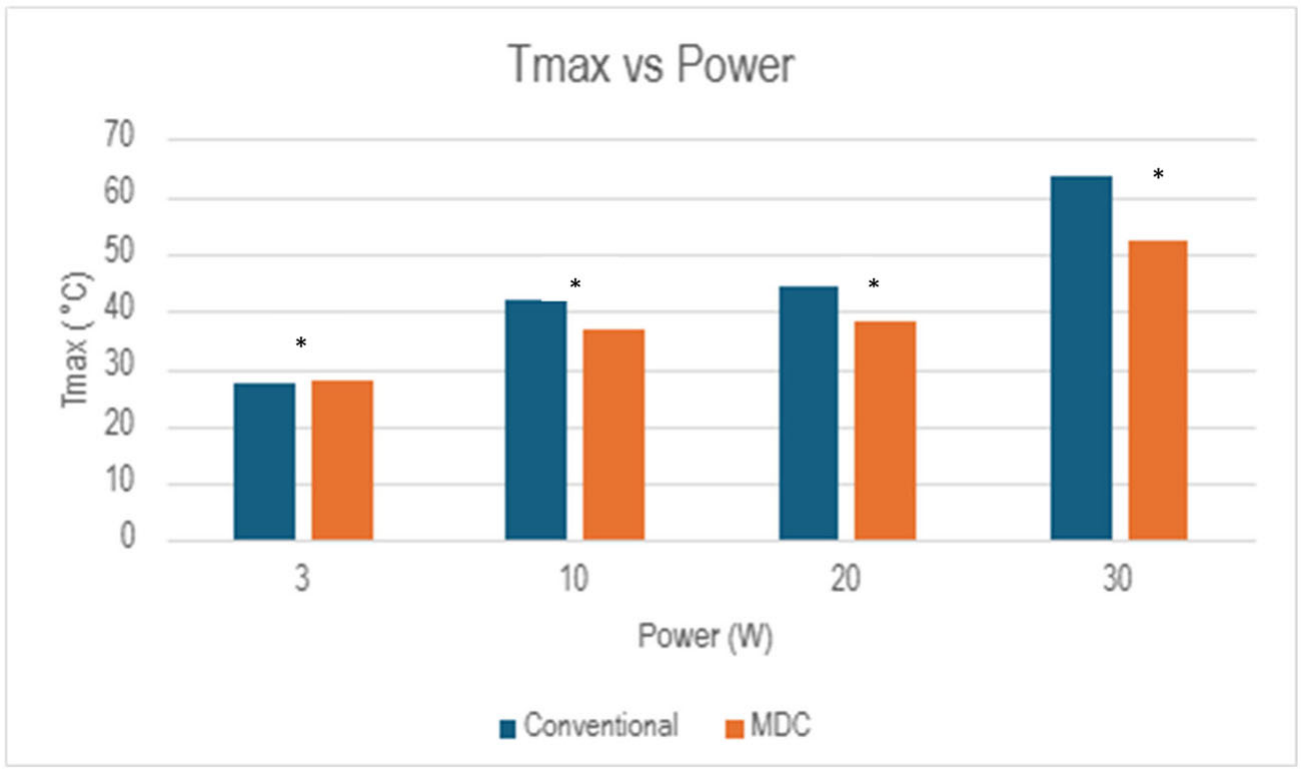
Comparison of maximum temperature at each power setting. *p < 0.05.

At the 3 W setting, the Tmax for both lasers did not surpass 30°C (Figure 2a). The MDC TFL reached a Tmax of 27.7°C, while the conventional TFL reached a Tmax of 27.3°C (*p* < 0.05, Figure 4). Both lasers reached Tmax at the same time (30 s). Both the MDC and conventional TFL did not surpass 43°C when operating at 3 W (Figure 3a).

At 10 W, the MDC TFL demonstrated a significantly lower Tmax of 36.9°C, compared to a Tmax of 42.1°C observed in the conventional TFL (*p* < 0.01, Figure 2b). The MDC TFL reached Tmax after 48 s, while the conventional TFL reached Tmax after 54 s. Despite this difference, neither laser exceeded 43°C at the 10 W settings (Figure 3b).

At 20 W, the MDC TFL showed a lower Tmax of 38.1°C compared to the conventional TFL which reached a Tmax of 44.6°C (*p* < 0.01, Figure 2c). The MDC TFL reached Tmax after 54 s, while the conventional TFL reached Tmax after 48 s. Although the MDC TFL did not exceed 43°C at the 20 W setting, the CEM₄₃ for the conventional TFL reached 0.9 equivalent minutes (Figure 3c).

At 30 W, the temperature difference was greatest between the two lasers (Figure 3). The MDC TFL reached a Tmax of 53.6°C, whereas the conventional TFL reached a Tmax of 63.6°C (*p * < 0.05, Figure 2d). The MDC TFL reached Tmax at 18 s, while the conventional TFL reached Tmax at 42 s. Additionally, while the MDC TFL did not exceed 60°C, the conventional TFL surpassed this threshold for nearly 30 s. Furthermore, the CEM₄₃ revealed a difference in the overall thermal load between the two lasers. The CEM₄₃ for the MDC TFL reached 425 equivalent minutes, compared to 275,919 equivalent minutes for the conventional TFL (Figure 3d).

## 4 Discussion

In this experiment, five trials of 60 s continuous activation were conducted in four different power settings, (3 W, 10 W, 20 W, and 30 W) for both TFL versions. The MDC TFL generated lower temperatures than the Soltive TFL at the 10 W, 20 W, and 30 W settings. The CEM₄₃ values did not exceed the threshold of 20 equivalent minutes at the 3 W, 10 W, and 20 W settings for either laser. However, at 30 W, both lasers generated significant heat above the threshold for thermal injury (CEM₄₃ > 20), with the Soltive TFL generating 649 times greater CEM₄₃ than the MDC TFL. These findings are consistent with the thermodynamics of laser activation where higher power settings generate higher temperatures [8]. The MDC TFL produced less heat in the 10 W, 20 W, and 30 W settings, emphasizing that laser duty cycle modification effectively reduced heat generation during laser activation.

As RIRS emerges as the most common surgical modality for kidney and ureteral stone treatment, a thorough understanding of laser principles is warranted. The concept of laser duty cycle provides a critical insight into how laser design alternation can reduce heat generation during activation. The duty cycle is defined as the percentage of time the laser remains active within a complete on/off cycle and is calculated by dividing the pulse width (the duration the laser is on in one cycle) by the pulse period (the total time of one on/off cycle). There are three types of duty cycles: an intrinsic duty cycle (IDC), an operative duty cycle (ODC), and a procedural duty cycle (PDC). IDC is generated based on the energy settings and changes specifically within the pulse width of the laser configured by the manufacturer, while ODC is the percentage of time that the laser is actually firing during a full activation cycle. The procedural duty cycle (PDC) that is sometimes used interchangeably with ODC, though they represent distinct entities, is the percentage of time that the surgeon is stepping on the pedal divided by total time that the laser is being used [9]. A higher duty cycle percentage corresponds to a longer pulse width, less off time between pulses, greater energy delivery and, consequently, increased heat generation. Based on laser physics, shorter pulse widths generate high peak temperatures with lower cumulative heat, whereas longer pulses produce lower peak temperatures but higher cumulative heat.

**Figure 5:**
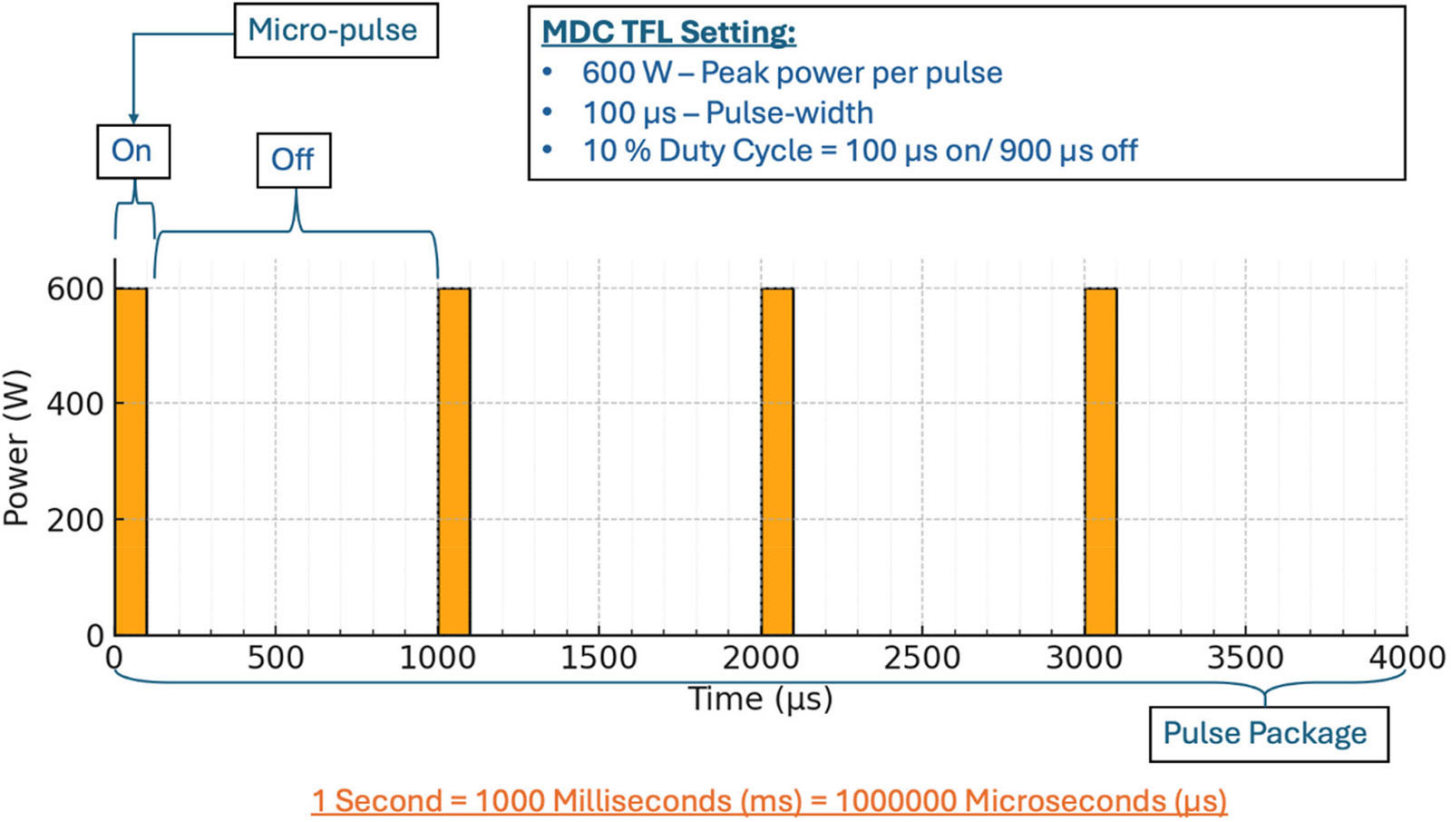
Graphic demonstration of the activation pulse package concept.

The conventional TFL operates with longer pulse widths of 1000–2000 µs, resulting in greater cumulative heat and a higher risk of thermal injury. In contrast, the MDC TFL features adjustable pulse‐widths of 100 and 500 µs, optimized to minimize heat buildup and subsequent tissue injury. The MDC TFL operates using a pulse modulation system that employs an activation pulse package concept. Laser peak powers are 500 W per pulse for 500 µs pulse‐width, and 600 W per pulse for 100 µs pulse‐widths. Within each activation pulse package, a variable number of micro‐pulses determined by a propriety software algorithm that is dependent upon energy setting (J) that is selected by the surgeon. When the surgeons select the frequency (Hz) the machine will apply that number of activation pulse packages per second. A wider pulse‐width of 500 µs helps reduce stone retropulsion and allows for effective coagulation when used for tissue ablation [10]. When the machine is operated in 100 µs pulse‐width the laser is activated for 100 µs followed by 900 µs pause (Figure 5). This ratio creates a 10% intrinsic duty cycle. The number of 100 µs activations within the pulse package is determined by the energy setting selected with higher energy requiring more activation pulses per package. In contrast, when operated at 500 µs pulse width, each micro‐pulse is 500 µs, followed by a 500 µs pause resulting in an intrinsic duty cycle of 50%. As both pulse energy and repetition rate increase, the duty cycle increases generating higher energy and elevating tissue temperature.

The thermal effects and temperature dynamics during lithotripsy have been extensively studied for both the HL and TFL platforms. Several factors influence temperature regulation during laser lithotripsy. Laser type is critical, with the TFL generally generating higher temperatures compared to the HL [3]. Laser power also plays a central role, as higher power settings directly increase temperatures, regardless of the energy‐to‐frequency ratio [11]. In addition to laser energy and frequency settings, the surgeon's activation behavior, i.e., PDC, significantly impact thermal load during the procedure. Thermal dose is influenced by fiber‐to‐target distance [12], irrigation rate and temperature [3], and use of a ureteral access sheath (UAS), which enhances outflow and cooling [9]. Finally, stone location and composition play a key role in heat generation during laser lithotripsy. Limited fluid movement in confined spaces like the ureter or behind stenotic infundibula reduces heat dissipation [8], while harder stones require higher energy and longer laser irradiation times.

This study is the first to evaluate the role of MDC on heat generation trends, comparing an MDC TFL to a conventional TFL. Our findings introduce a modified internal duty cycle as a potential new factor decreasing heat generation during laser lithotripsy, addressing a gap in the current literature. Given the critical impact of heat management during RIRS, these results emphasize the importance of considering MDC when evaluating TFL platforms or developing new laser systems. This new approach could significantly enhance procedural safety by providing better thermal control, ultimately improving patient outcomes.

This study has several limitations. While the reduced heat generation of the MDC TFL offers a clear advantage in mitigating thermal injury, the effects of this MDC on fragmentation efficiency need further research. As a benchtop model, this study may not perfectly replicate all factors encountered during clinical lithotripsy. As this experiment focused on thermal safety, laser fragmentation efficiency was not assessed. Additionally, this study compared the MDC TFL to only one other thulium laser technology. Future studies could compare the MDC TFL to other thulium lasers.

## 5 Conclusion

The TFL with a modified laser duty cycle showed a significant reduction in intraluminal heat generation and a significant difference in CEM₄₃, compared to the conventional TFL when operated at high power settings. Understanding the effects of duty cycle on heat generation during laser lithotripsy could improve safety. The MDC TFL is a promising option for reducing risk of thermal injury.

## Ethics Statement

The authors have nothing to report.

## Consent

The authors have nothing to report.
